# Medicare Advantage enrollment and outcomes of post-acute nursing home care among patients with dementia

**DOI:** 10.1093/haschl/qxae084

**Published:** 2024-06-13

**Authors:** Daeho Kim, David J Meyers, Laura M Keohane, Hiren Varma, Emma M Achola, Amal N Trivedi

**Affiliations:** Department of Health Services, Policy and Practice, Brown University, Providence, RI 02903, United States; Department of Health Services, Policy and Practice, Brown University, Providence, RI 02903, United States; Department of Health Policy, Vanderbilt University, Nashville, TN 37203, United States; Department of Health Services, Policy and Practice, Brown University, Providence, RI 02903, United States; Department of Health Policy, Vanderbilt University, Nashville, TN 37203, United States; Department of Health Services, Policy and Practice, Brown University, Providence, RI 02903, United States; Providence VA Medical Center, Providence, RI 02908, United States

**Keywords:** Medicare Advantage, post-acute care, dementia

## Abstract

Enrollment in Medicare Advantage (MA) has been rapidly growing. We examined whether MA enrollment affects the outcomes of post-acute nursing home care among patients with Alzheimer's disease and related dementias (ADRD). We exploited year-to-year changes in MA penetration rates within counties from 2012 through 2019. After adjusting for patient-level characteristics and county fixed effects, we found that MA enrollment was not associated with days spent at home, nursing home days, likelihood of becoming a long-stay resident, hospital days, hospital readmission, or 1-year mortality. There was a modest increase in successful discharge to the community by 0.73 percentage points (relative increase of 2.4%) associated with a 10-percentage-point increase in MA enrollment. The results are consistent among racial/ethnic subgroups and dual-eligible patients. These findings suggest an imperative need to monitor and improve quality of managed care among enrollees with ADRD.

## Introduction

As of March 2023, more than half of all Medicare beneficiaries were enrolled in Medicare Advantage (MA) plans—private managed-care plans—increasing from 29% in 2013 to 51% in 2023.^1^ Unlike traditional Medicare (TM), MA plans receive capitated payments to cover all services rendered to their enrollees, with bonus payments based on their quality of care, which incentivizes plans to reduce costs and possibly to improve care quality. In addition, most MA plans offer reduced cost-sharing as well as out-of-pocket spending limits and supplemental benefits that are unavailable in TM.^2^ These features of MA plans are highly attractive to high-need, high-cost Medicare beneficiaries, such as those with Alzheimer's disease and related dementias (ADRD).^3^ However, MA plans’ utilization control strategies, such as restrictive provider networks and prior authorization requirements, can also create challenges for such patients if they reduce access to care^4-6^ or to high-quality providers.^7,8^

More than 6 million Americans aged 65 years and older were living with ADRD in 2023, about half of whom are nursing home residents.^9,10^ The prevalence of ADRD is expected to continue to grow to 14 million by 2060.^9^ Persons with ADRD have substantially higher morbidity, mortality, and health care spending than those without ADRD.^11-15^ Alzheimer's disease and related dementias were the fifth-leading cause of death among elderly persons in 2019 and accounted for $345 billion in spending in 2023. Nearly half of these expenditures are paid by Medicare, with nursing home care (including post-acute and long-term services) accounting for the highest per-person spending.^9^

Despite rapidly growing MA penetration and increasing prevalence of ADRD, especially among nursing home residents, there is limited evidence on the impacts of MA on the outcomes of post-acute nursing home care among patients with ADRD. Most prior research on the care utilization and outcomes of patients with ADRD is based on those enrolled in TM, with remarkably few studies on those enrolled in MA plans.^8,16^ The primary empirical challenge in estimating the impacts of MA on persons with ADRD is favorable selection arising from MA plans attracting healthier enrollees.^17-20^

In this study, we exploit within-county changes in the MA penetration rate, which is less vulnerable to favorable selection. We examined the association of MA enrollment with the outcomes of post-acute nursing home care among patients with ADRD aged 65 years and older who were admitted to a nursing home following an acute-care hospital discharge between 2012 and 2019.

## Data and methods

### Data sources and study population

The primary data sources were 100% of the Centers for Medicare and Medicaid (CMS) Master Beneficiary Summary File (MBSF), Medicare Provider and Analysis Review (MedPAR) file, Minimum Data Set (MDS), the Residential History File (RHF),^21^ and publicly available Nursing Home Compare star ratings.

The MBSF was used to identify the Medicare beneficiary's MA or TM status at the time of a nursing home index admission. The MBSF also contains a beneficiary's demographic characteristics such as age, sex, race/ethnicity, and dual eligibility for Medicare and Medicaid. The MedPAR file contains hospital inpatient claims for both MA and TM enrollees. For MA enrollees, the MedPAR includes claims for those admitted to hospitals that receive disproportionate share hospital (DSH) payments or graduate medical education (GME) payments. A previous study showed that the MedPAR covers 92% of all MA hospitalizations in those hospitals receiving DSH or GME payments.^22^ We used MedPAR to identify post-acute nursing home residents who had an acute-care hospitalization within 7 days prior to a nursing home index admission. The MedPAR was also used to measure 30-day hospital readmission following the nursing home index admission.

The MDS data provide detailed information for every nursing home resident on diagnoses, physical, emotional, and cognitive functioning, including activities of daily living (ADL) score and Brief Interview for Mental Status (BIMS), a basis of the Cognitive Function Scale (CFS) score.^23^ The RHF summarizes information from Medicare claims and assessments to track Medicare beneficiaries through health care settings, such as hospital, nursing home, inpatient rehabilitation facility, and home health agency.^21^ The Nursing Home Compare star ratings—ranging from 1 to 5—measure the overall quality of a nursing home.^24^ We linked all data at the beneficiary level.

For both MA and TM enrollees, we identified an individual with ADRD if a diagnosis of Alzheimer's disease or non-Alzheimer's dementia (eg, Lewy body dementia) was reported by the nursing home in MDS data or if the CFS score indicated moderate or severe impairment (scores 3 or 4). In sensitivity analyses, we used different definitions of ADRD: one based on the aforementioned diagnosis only and the other based on the CFS score only.

The final study population included 3 358 230 Medicare beneficiaries with ADRD admitted to a nursing home between January 1, 2012, and December 31, 2019, directly from an acute-care hospital discharge with less than 90 days of nursing home care in the preceding year. We excluded persons who were long-stay nursing home residents before their index admission because such individuals would be unlikely to return to the community and would be expected to continue to reside in a nursing home.

### Measures

The primary outcomes were days spent at home, nursing home days, likelihood of becoming a long-stay nursing home resident, hospital days, unplanned 30-day hospital readmission, successful discharge to the community, and 1-year mortality rate. The secondary outcome was the likelihood of admission to a high-quality nursing home as a possible mechanism through which MA enrollment affects the primary outcomes. We define the days spent at home as the number of days alive subtracting total number of nursing home and hospital days within 365 days following the nursing home index admission. Nursing home days and hospital days were calculated within 365 days from the index admission. The likelihood of becoming a long-stay resident is a binary indicator for a nursing home resident who spent more than 100 days in the nursing home following the index admission to the nursing home. For unplanned hospital readmission within 30 days of the index admission, we applied the CMS algorithm.^25^ Successful discharge to the community is measured by a discharge from nursing home to the community within 100 days of the index admission without subsequent nursing home admission, hospital readmission, or death in 30 days following the nursing home discharge. The 1-year mortality rate is calculated based on a death occurring within 365 days of the index admission. High-quality nursing homes were defined as nursing homes with an overall star rating of 4 or 5.

The primary explanatory variable of interest was a year-to-year change in MA penetration within counties among nursing home residents with ADRD. Other covariates included age (grouped into 5-year age bins), dual eligibility for Medicare and Medicaid, ADL and CFS scores from MDS data, as well as variables reflecting characteristics of the preceding hospital stay: use of an intensive care unit (ICU), hospital length of stay (LOS), and Elixhauser comorbidity index.^26^

### Study design

We exploited year-to-year changes in the MA penetration rate within counties from 2012 through 2019, as used in another study.^27^ This approach compares changes in outcomes of all Medicare beneficiaries within the same county as the proportion of MA enrollment changes in the county, rather than comparing outcomes for MA vs TM enrollees, which is known to be subject to selection bias. Our approach is more robust to favorable selection because the selection (measured by difference in risk scores between MA and TM, for example) occurs only at the county level (ie, MA market level). Thus, the sociodemographic and clinical characteristics including risk score of both MA and TM enrollees within a county should not change over time, except through movement of enrollees across counties or through death. We empirically tested this assumption in sensitivity analyses described below. In addition, our approach is robust to MA plans’ strategic selection in choosing counties to enter/exit, which affects county-level MA penetration, because it is unlikely that the plans can predict future improvements in outcomes for patients with ADRD in a given county, beyond the plan's own potential impact on those outcomes.

### Statistical analysis

We used multivariable linear regression models for year-to-year changes in county-level MA penetration and the outcomes of post-acute nursing home care from 2012 through 2019 (Appendix A1). All estimates were scaled by a 10-percentage-point (-pp) increase in MA penetration rate, which is the sample mean of the county-level changes in MA penetration from 2012 to 2019. All models were adjusted for the beneficiary's demographic and clinical characteristics (age, dual eligibility for Medicare and Medicaid, ADL score, CFS score, Elixhauser comorbidity index, and the use of ICU and the LOS in the discharged hospital prior to the index admission) as well as county and year fixed effects. Heteroscedasticity-adjusted standard errors were clustered at the county level to account for serial correlation within counties in an unrestricted way. Given prior evidence that enrollment in MA varies by race, ethnicity, and dual Medicare–Medicaid status,^28^ we stratified the main analyses by patients' race/ethnicity (non-Hispanic White, non-Hispanic Black, and Hispanic) and dual-eligible status.

### Sensitivity analysis

First, as a validity check for the underlying assumption of our regression specification, we estimated the regression models for each beneficiary's clinical characteristics (ADL score, CFS score, ICU use, and hospital LOS) as an outcome variable to test if it was associated with changes in MA penetration. Second, to assess whether the composition of post-acute patients with ADRD changed differentially across counties with varying growth in MA penetration, we examined the following additional outcomes: the log number of post-acute patients with ADRD and the Elixhauser comorbidity index of those patients. Third, we conducted analyses applying different ways of identifying beneficiaries with ADRD: one based on diagnoses only and the other based on CFS score only to assess whether the results of the main analyses were driven by the method of identifying ADRD.

### Limitations

This study has several limitations. First, our findings only apply to patients with ADRD who received post-acute nursing home care, and thus may not extend to other Medicare populations with different conditions in different care settings.

Second, the study data used to identify post-acute patients and to calculate 30-day hospital readmission, the MedPAR files, do not cover all hospitalizations for MA enrollees, although they cover over 90% of all MA hospitalizations in hospitals receiving DSH or GME payments.^22,29^ However, as long as the coverage rate of MedPAR for MA hospitalization did not change over time differentially across counties so that changes in the coverage rate were uncorrelated with changes in MA penetration rates, our results would be unbiased.

Third, prior studies have identified that, relative to TM, MA plans inflate the number of coded diagnoses among their enrollees because payments to these plans are risk-adjusted on the basis of the number of comorbid conditions.^27,30,31^ However, our findings are unlikely to be driven by incentives for MA plans to inflate the number of comorbid conditions or to overcode a diagnosis of dementia for 2 reasons: first, we relied on assessments from nursing homes to identify dementia, cognitive impairment, and limitations in ADL, which are not used to derive payment rates; and second, dementia was not included as one of the Hierarchical Condition Categories—a basis of the risk-adjusted payments—during the study period.

Fourth, to the extent that MA plans can selectively identify and enter counties in which future nursing home outcomes improve, that would bias the findings towards better outcomes in counties with high-growth in MA penetration.

Finally, we cannot fully exclude the possibility that other county-level changes in unobserved factors may have accounted for our results. However, our finding of no correlation between changes in MA penetration and changes in observed patients’ clinical characteristics makes this possibility less likely.

## Results

The study population of 3 358 230 Medicare beneficiaries included 822 410 (24.5%) MA enrollees (mean [SD] age, 83.3 [7.6] years; 13.6% non-Hispanic Black; 8.2% Hispanic; 33.8% dual-eligible for Medicaid) and 2 535 820 (75.5%) TM enrollees (mean [SD] age, 83.9 [7.8] years; 10.7% non-Hispanic Black; 5.3% Hispanic; 34.3% dual-eligible for Medicaid) who were diagnosed with ADRD and admitted to a nursing home between January 1, 2012, and December 31, 2019 (Table 1). The MA penetration rate increased from 19.3% in 2012 to 29.9% in 2019 (Appendix Figure A1), with substantial county-level variation in changes in MA penetration rate from 2012 to 2019 (mean: 10 percentage points; IQR, 0.4 to 16.5) (Appendix Figure A2).

**Table 1.** Characteristics of Medicare beneficiaries with ADRD, 2012 to 2019.

	MA	TM	Difference (MA–TM)
*n*	822 410	2 535 820	3 358 230
Age (SD), y	83.3 (7.6)	83.9 (7.8)	−0.6***
Female, %	61.8	62.7	−0.9***
White, %	74.6	80.7	−6.0***
Black, %	13.6	10.7	2.9***
Hispanic, %	8.2	5.3	2.9***
Dual eligibility, %	33.8	34.3	−0.5***
ADL score	19.2 (4.1)	19.4 (4.5)	−0.1***
CFS score	2.6 (0.9)	2.6 (0.9)	−0.0***
ICU use,^a^ %	28.7	27.2	1.5***
Hospital LOS,^a^ days	7.8 (8.8)	7.6 (12.1)	0.2***
Elixhauser index	4.1 (2.0)	4.0 (2.0)	0.1***

Abbreviations: ADRD, Alzheimer's disease and related dementias; ADL, activities of daily living; CFS, Cognitive Function Scale; ICU, intensive care unit; LOS, length of stay; MA, Medicare Advantage; TM, Traditional Medicare.

^***^ *P* < .01.

^a^ICU use and hospital LOS in a discharged hospital prior to index nursing home admission.

Appendix Figure A3 plots associations between county-level changes in MA penetration and the outcomes. The figure shows virtually no association, except for successful discharge to the community (panel F). In Table 2, the regression estimates confirm these patterns. In adjusted analyses, we found that an increase in MA penetration was not associated with days spent at home (estimate: 1.02; 95% CI: −0.19 to 2.23), nursing home days (0.12; 95% CI: −1.24 to 1.49), likelihood of becoming a long-stay resident (−0.04 pp; 95% CI: −0.47 to 0.39), hospital days (−0.05; 95% CI: -0.12 to 0.2), 30-day hospital readmission (−0.16 pp; 95% CI: −0.36 to 0.03), and 1-year mortality (−0.16 pp; 95% CI: −0.56 to 0.24). There was a modest increase in successful discharge to the community by 0.73 pp (95% CI: 0.33 to 1.12), a relative increase of 2.42% (95% CI: 1.10% to 3.72%). These findings can be explained, in part, by no association between MA enrollment and admission to high-quality nursing homes (−0.41 pp; 95% CI: −0.96 to 0.14). The results were consistent in stratified analyses of racial/ethnic minority groups (Table 3) and dual-eligible patients (Table 4).

**Table 2.** Outcomes of post-acute nursing home care associated with a 10-percentage-point increase in Medicare Advantage penetration in a county.

	Sample mean	Unadjusted (95% CI)	Adjusted (95% CI)
Days spent at home	110.53	1.43 (0.13 to 2.73)	1.02 (−0.19 to 2.23)
Nursing home days	135.62	0.05 (−1.38 to 1.49)	0.12 (−1.24 to 1.49)
Long-stay NH resident (%)	37.85	−0.07 (−0.53 to 0.39)	−0.04 (−0.47 to 0.39)
Hospital days	4.35	−0.03 (−0.10 to 0.04)	−0.05 (−0.12 to 0.02)
Hospital readmission (%)	8.03	−0.13 (−0.33 to 0.06)	−0.16 (−0.36 to 0.03)
Successful discharge to community (%)	30.11	0.81 (0.39 to 1.24)	0.73 (0.33 to 1.12)
One-year mortality (%)	44.29	−0.30 (−0.70 to 0.10)	−0.16 (−0.56 to 0.24)
Admission to NH with star rating ≥4 (%)	48.87	−0.44 (−1.00 to 0.11)	−0.41 (−0.96 to 0.14)

Abbreviations: ADL, activities of daily living; CFS, Cognitive Function Scale; ICU, intensive care unit; LOS, length of stay; MA, Medicare Advantage; NH, nursing home.

All estimates are scaled by a 10-percentage-point increase in MA penetration in a county. Adjusted for age, race, dual eligibility for Medicare and Medicaid, ADL score, CFS score, Elixhauser comorbidity index, the use of ICU and hospital LOS in a discharged hospital prior to the NH index admission, as well as county and year fixed effects. Standard errors are clustered at the county level.

**Table 3.** Outcomes of post-acute nursing home care by race/ethnicity associated with a 10-percentage-point increase in Medicare Advantage penetration in a county.

	Hispanic		Non-Hispanic Black		Non-Hispanic White	
	Estimate	(95% CI)	Estimate	(95% CI)	Estimate	(95% CI)
Days spent at home	1.93	(−3.02 to 6.87)	1.75	(−0.80 to 4.29)	0.58	(−0.64 to 1.80)
Nursing home days	−1.99	(−7.00 to 3.01)	0.11	(−2.81 to 3.02)	0.29	(−1.12 to 1.69)
Long-stay NH resident (%)	−0.88	(−2.57 to 0.81)	−0.24	(−1.19 to 0.71)	0.05	(−0.41 to 0.50)
Hospital days	−0.33	(−0.75 to 0.10)	−0.06	(−0.33 to 0.20)	−0.06	(−0.13 to 0.01)
Hospital readmission (%)	−0.78	(−1.85 to 0.29)	−0.16	(−0.74 to 0.42)	−0.10	(−0.30 to 0.10)
Successful discharge to the community (%)	0.51	(−1.11 to 2.13)	0.81	(−0.06 to 1.67)	0.51	(0.11 to 0.91)
One-year mortality (%)	−0.08	(−1.64 to 1.49)	−0.03	(−0.99 to 0.93)	−0.08	(−0.50 to 0.34)
Admission to NH with star rating ≥4 (%)	1.17	(−3.39 to 1.06)	0.38	(−1.05 to 1.81)	−0.52	(−1.08 to 0.04)

Abbreviations: ADL, activities of daily living; CFS, Cognitive Function Scale; ICU, intensive care unit; LOS, length of stay; MA, Medicare Advantage; NH, nursing home.

All estimates are scaled by a 10-percentage-point increase in MA penetration in a county. Adjusted for age, dual eligibility for Medicare and Medicaid, ADL score, CFS score, Elixhauser comorbidity index, the use of ICU and hospital LOS in a discharged hospital prior to the NH index admission, as well as county and year fixed effects. Standard errors are clustered at the county level.

**Table 4.** Outcomes of post-acute nursing home care by dual-eligibility status associated with a 10-percentage-point increase in Medicare Advantage penetration in a county.

	Duals		Non-duals	
	Estimate	(95% CI)	Estimate	(95% CI)
Days spent at home	1.38	(0.12 to 2.65)	0.73	(−0.61 to 2.07)
Nursing home days	−0.16	(−1.78 to 1.46)	0.62	(−0.91 to 2.14)
Long-stay NH resident (%)	−0.03	(−0.54 to 0.47)	0.10	(−0.39 to 0.58)
Hospital days	−0.06	(−0.15 to 0.04)	−0.00	(−0.08 to 0.07)
Hospital readmission (%)	−0.28	(−0.56 to 0.00)	−0.13	(−0.34 to 0.08)
Successful discharge to community (%)	0.65	(0.26 to 1.03)	0.74	(0.32 to 1.17)
One-year mortality (%)	−0.19	(−0.70 to 0.32)	−0.22	(−0.66 to 0.22)
Admission to NH with star rating ≥4 (%)	−0.33	(−1.06 to 0.40)	−0.40	(−0.98 to 0.19)

Abbreviations: ADL, activities of daily living; CFS, Cognitive Function Scale; ICU, intensive care unit; LOS, length of stay; MA, Medicare Advantage; NH, nursing home.

All estimates are scaled by a 10-percentage-point increase in MA penetration in a county. Adjusted for age, race, ADL score, CFS score, Elixhauser comorbidity index, the use of ICU and hospital LOS in a discharged hospital prior to the NH index admission, as well as county and year fixed effects. Standard errors are clustered at the county level.

Appendix Figure A4 and Appendix Table A1 show the results of the validation test for the regression estimation: changes in the beneficiary's clinical characteristics (measured at the time of admission to a nursing home) are uncorrelated with changes in MA penetration. The results support the validity of our approach. Appendix Table A2 shows no evidence that the composition of post-acute patients with ADRD changed over time differentially between counties with higher vs lower growth in MA enrollment. In addition, the findings were consistent in analyses that identify beneficiaries with ADRD based on diagnoses only and based on CFS score only (Appendix Table A3).

## Discussion

We found that increases in MA enrollment were not associated with improved outcomes of post-acute care among patients with ADRD, except for a modest increase in successful discharge to the community. The results were consistent in stratified analyses of racial/ethnic minorities and dual-eligible patients, despite their disproportionately high enrollment in the MA program relative to their counterparts.^28^ The findings were robust to the method of identifying enrollees with ADRD using either a diagnostic code for ADRD or a measure of cognitive function from the nursing home admission assessment. The lack of a meaningful association between MA enrollment and improved outcomes was observed despite a 10-percentage-point increase (or 50% relative increase) in MA enrollment during the 2012–2019 study period. To our knowledge, this is the first national study of the outcomes of nursing home care for patients with ADRD in the MA program.

It is noteworthy that our estimates include spillover effects of MA on TM enrollees in post-acute nursing home care as we exploited county-level variation in MA growth. For example, if nursing homes changed their delivery of care to adapt to increased MA enrollment, those changes might affect both MA and TM enrollees in the same nursing homes in a county. Therefore, our estimates include the impacts of MA growth on both MA and TM enrollees in the same county.

Previous cross-sectional studies on different Medicare populations showed mixed findings. A recent study on general post-acute care showed that MA enrollees were less likely than TM enrollees to report improvement in functional status while using post-acute care but did not differ on other patient-reported outcomes,^32^ which is broadly consistent with our findings. In contrast, a study on hip fracture patients in nursing homes found that MA enrollees had lower hospital readmission rates and a lower likelihood of becoming a long-stay resident.^29^

There are 3 possibilities that may explain our findings. First, MA plans may not selectively contract with high-quality nursing homes for post-acute care for enrollees with ADRD. Indeed, consistent with previous work in the general Medicare population,^33^ we found no association between MA enrollment and admission to high-quality nursing homes. Another study showed that MA enrollment was associated with admission to low-quality nursing homes.^8^

Second, persons with ADRD and those using hospital and nursing home care have substantially high rates of disenrollment from MA to TM^34-36^; the exodus of high-cost, high-need populations out of MA plans may erode the potential benefits of managed care to improve outcomes for this population.

Third, our findings are consistent with evidence that persons with ADRD in MA plans are more likely to be excluded from routinely collected quality-measurement programs than MA enrollees without ADRD.^16^ Medicare Advantage plans may have fewer incentives to enhance quality for people with ADRD and have less insights into how to improve outcomes if performance measures exclude this population. Available survey data suggest that persons with ADRD in MA plans are more likely to report worse care experiences and difficulty accessing needed care.^16^

## Conclusion

Medicare Advantage enrollment was not associated with an improvement in the outcomes of post-acute nursing home care among Medicare beneficiaries with ADRD, except for a modest association with successful discharge to the community. The findings call into question whether continued expansion of managed care for high-cost, high-need populations such as those with ADRD will produce meaningful improvements in outcomes. The findings suggest an imperative need to monitor and improve the quality of managed care among enrollees with ADRD.

## Supplementary Material

qxae084_Supplementary_Data
