# Supplementary material for: Medicare Advantage enrollment and outcomes of post-acute nursing home care among patients with dementia
Source: Health Aff Sch. 2024 Jun 13;2(6):qxae084. doi: 10.1093/haschl/qxae084 (PMC11199989; doi:10.1093/haschl/qxae084)
Supplement: qxae084_Supplementary_Data [file qxae084_supplementary_data.zip › MA_ADRD_appendix_rev2_clean.docx]

**Appendix**

**Contents:**

**Appendix A1:** Regression specification

**Appendix Figure A1:** Change in MA penetration over time among persons with ADRD

**Appendix Figure A2:** Within-county change in MA penetration among persons with ADRD

**Appendix Figure A3:** Outcomes of Post-Acute Nursing Home Care

**Appendix Figure A4:** Graphical test for underlying assumption

**Appendix Table A1:** Test for underlying assumption

**Appendix Table A2:** Changes in the composition of post-acute nursing home patients with ADRD

**Appendix Table A3:** Outcomes of post-acute nursing home care associated with a 10-percentage point increase in Medicare Advantage penetration in a county using varying definitions of ADRD

**Appendix A1: Regression specification**

We estimated the following regression:

| $Y_{ict}=\gamma_{c}+\delta_{t}+\theta\cdot MA_{ct}+\beta X_{ict}+\varepsilon_{ict}$ | (e1) |
| --- | --- |

where $Y_{ict}$ is outcome of individual *i* in county *c* in time *t*; $\gamma_{c}$ are county fixed effects; $\delta_{t}$ are time fixed effects; $MA_{ct}$ is MA penetration rate in county *c* in time *t*; and $X_{ict}$ are time-varying individual-level covariates. The parameter of interest is θ that measures an association of MA enrollment with the outcome. Note that since the model includes county fixed effects, the regression essentially uses year-to-year changes in MA penetration within counties.

An underlying assumption for an unbiased estimate of *θ* in the regression model above is that year-to-year changes in MA penetration should not be correlated with the changes in unobserved factors that affect the outcome, which could be represented as follows:

| $Corr\left( \Delta MA_{ct},\Delta\varepsilon_{ict} \right)=0$ | (e2) |
| --- | --- |

Since we cannot directly observe those unobserved factors, $\varepsilon_{ict}$, we instead indirectly test if the changes in MA penetration is uncorrelated with changes in observed factors, $X_{ict}$:

| $Corr\left( \Delta MA_{ct},\Delta X_{ict} \right)=0$ | (e3) |
| --- | --- |

To test the correlation above, we estimated the following regression and test if $\lambda=0$:

| $X_{ict}=\gamma_{c}+\delta_{t}+\lambda\cdot MA_{ct}+\varepsilon_{ct}$ | (e4) |
| --- | --- |

where $X_{ict}$ is each of observed beneficiary clinical characteristics (ADL score, CFS score, ICU use, hospital LOS, and Elixhauser comorbidity index), and all other variables are the same as equation (e1).

Appendix Figure A1: Change in MA penetration over time among persons with ADRD

Appendix Figure A2: Within-county change in MA penetration among persons with ADRD


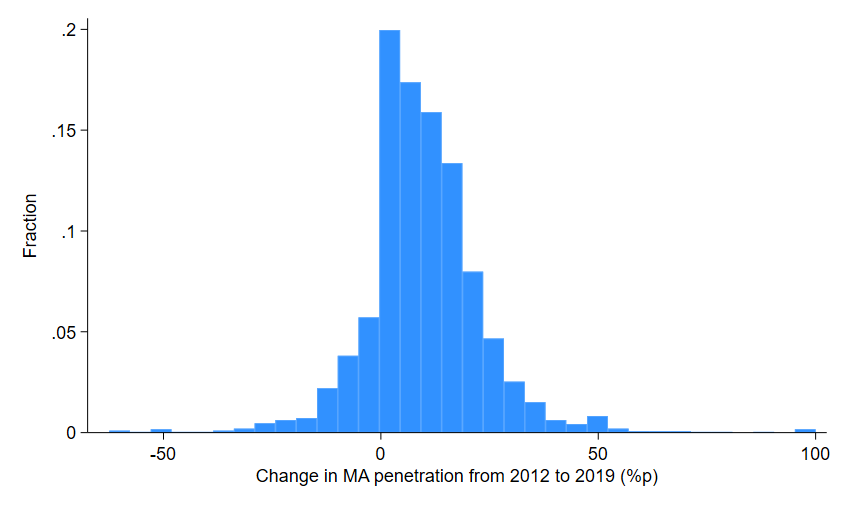


Appendix Figure A3: Outcomes of Post-Acute Nursing Home Care

A. Days spent at home


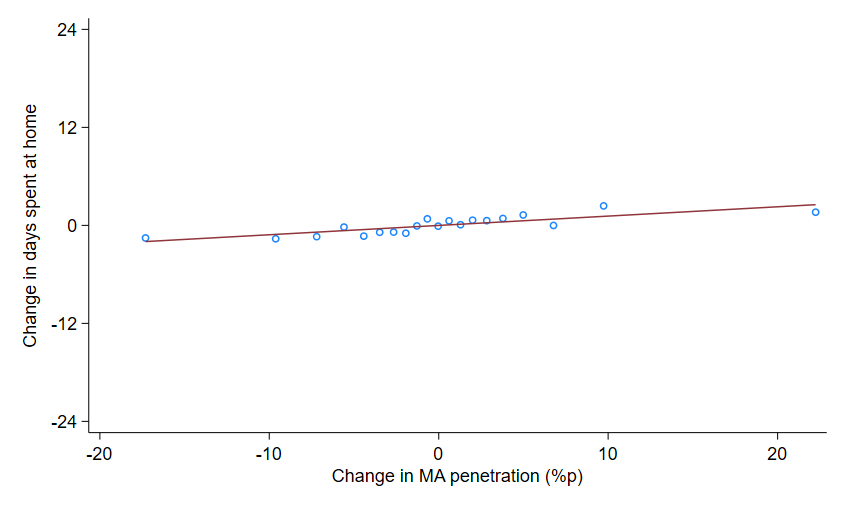


B. Nursing home days


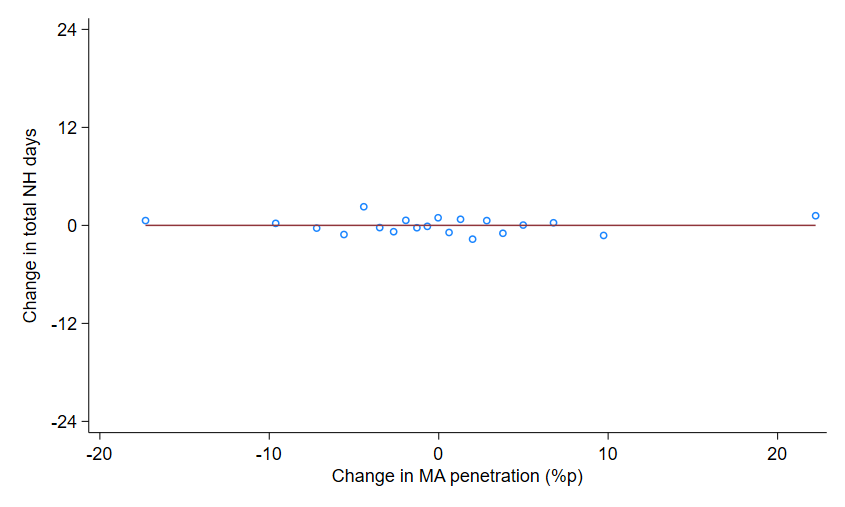


C. Becoming a long-stay resident


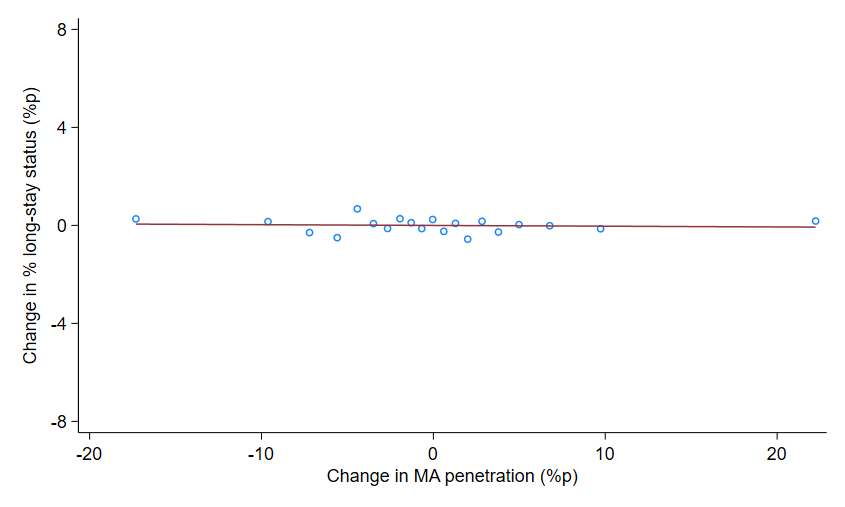


D. Hospital days


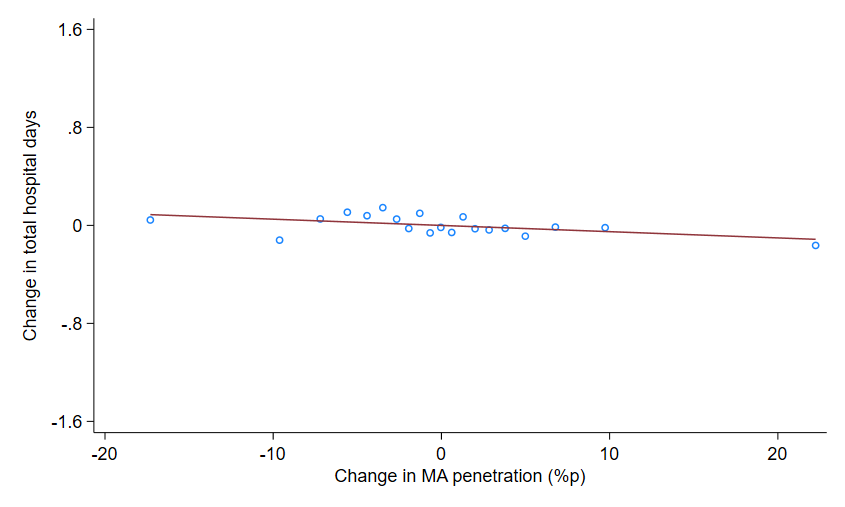


E. Hospital readmission


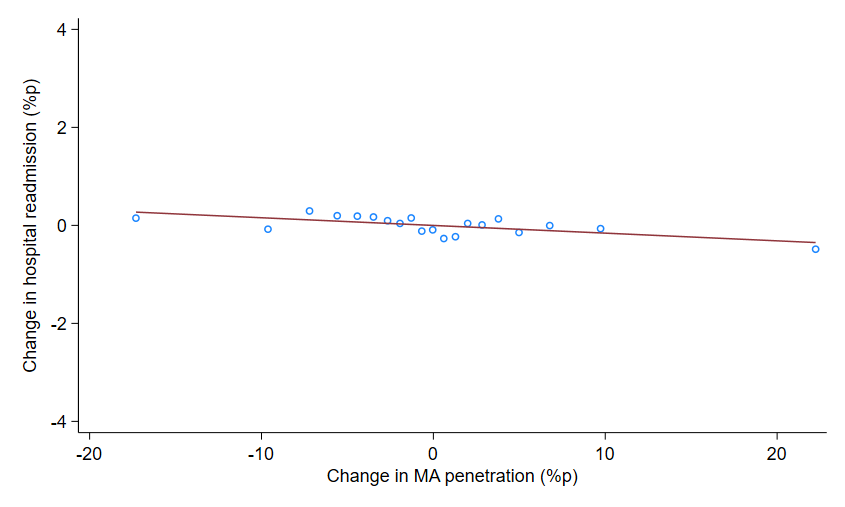


F. Successful discharge


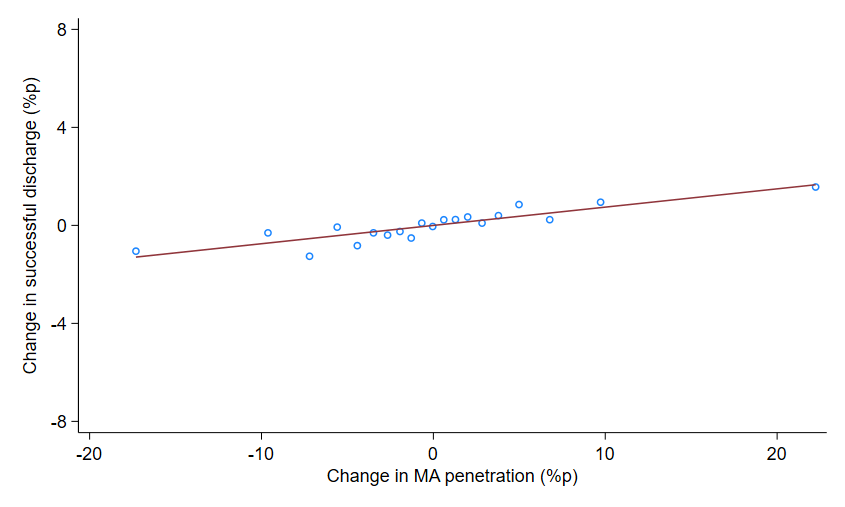


G. One-year mortality


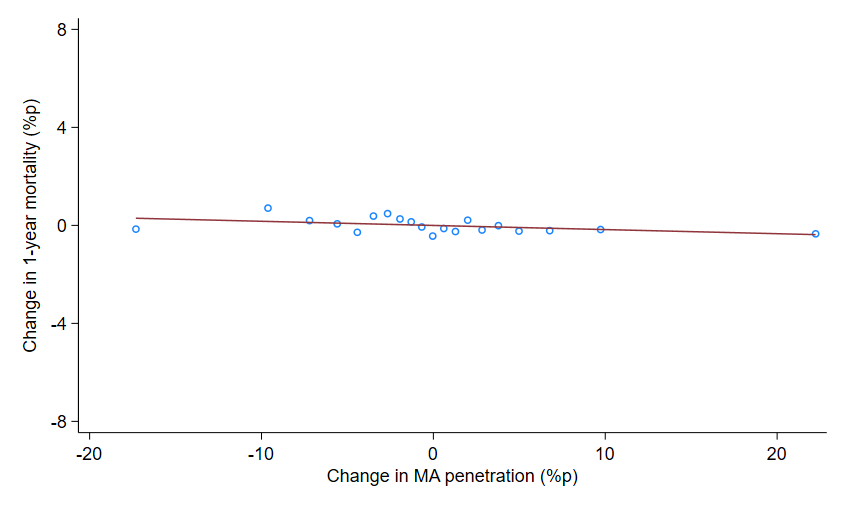


H. Percent admitted to high-quality nursing home


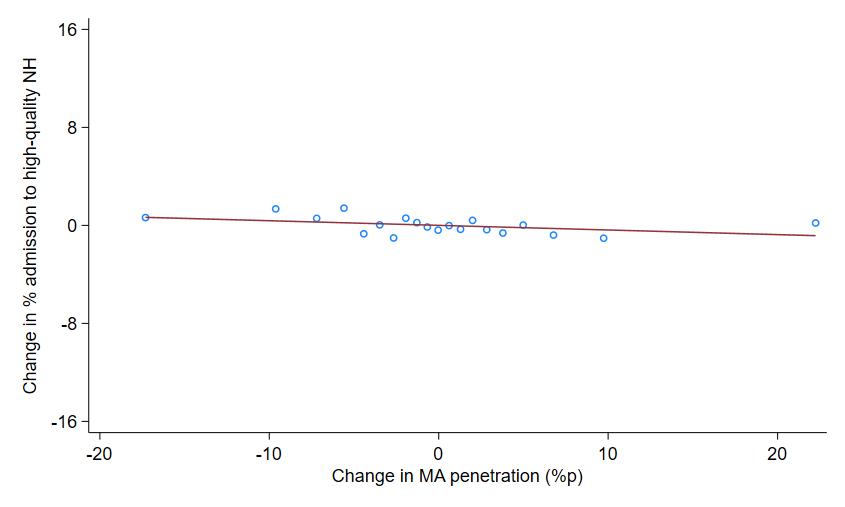


Note: Each panel shows a binned scatter plot, grouping counties into 20 equal-sized cells based on year-to-year changes in MA penetration rates from 2012 through 2019. Each dot represents a cell-level residualized mean from the regression estimation in equation (e1), with fitted line.

Appendix Figure A4: Graphical test for underlying assumption

A. Change in ADL score by change in MA penetration


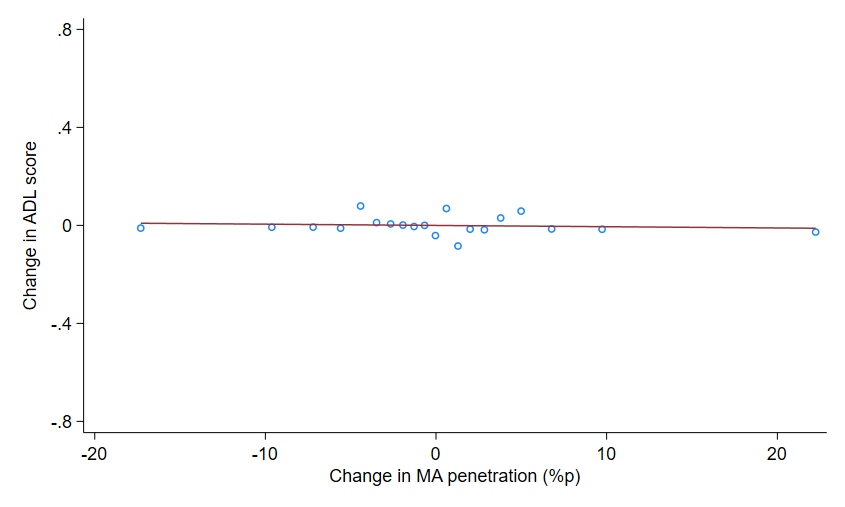


B. Change in CFS score by change in MA penetration


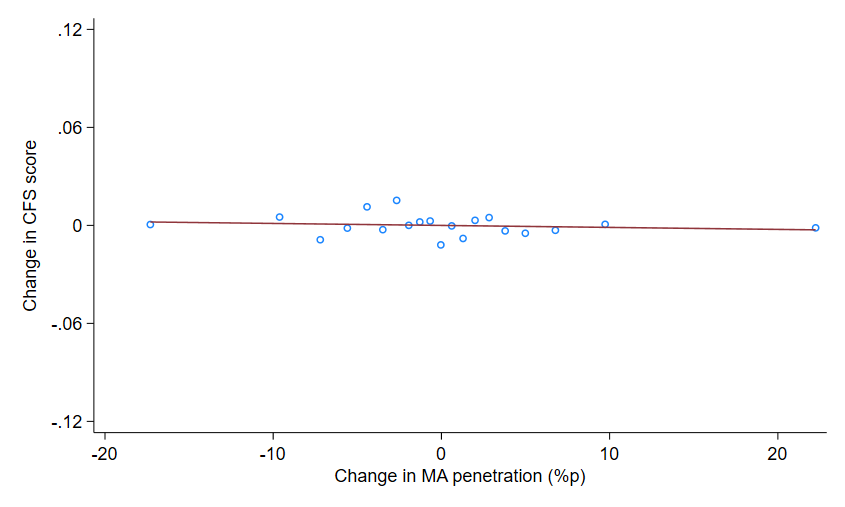


C. Change in ICU use by change in MA penetration


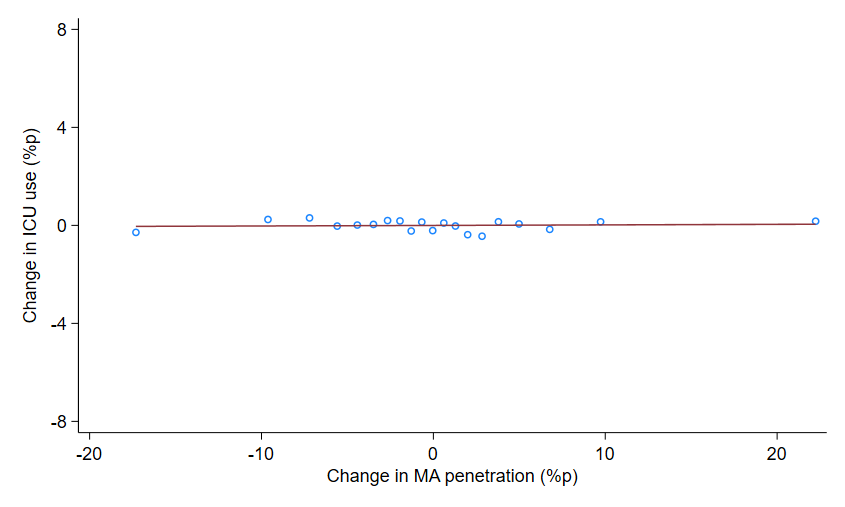


D. Change in hospital LOS by change in MA penetration


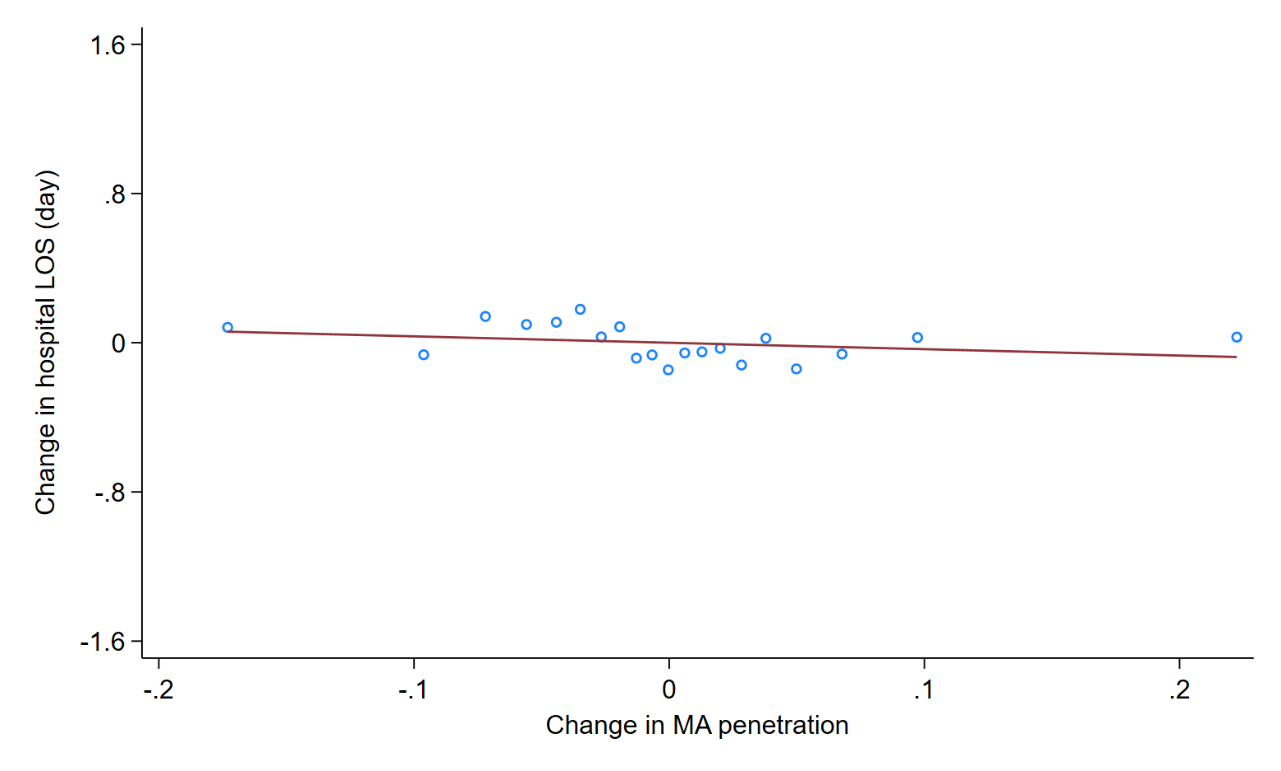


Notes: Each panel shows a binned scatter plot, grouping counties into 20 equal-sized cells based on year-to-year changes in MA penetration rates from 2012 through 2019. Each dot represents a cell-level residualized mean from the regression estimation in equation (e4), with fitted line.

Appendix Table A1: Test for underlying assumption

|  | Estimate (95% CI) | |
| --- | --- | --- |
| ADL score | -0.23 | (-0.69 to 0.22) |
| CFS score | -0.01 | (-0.08 to 0.06) |
| ICU use (%) | 1.17 | (-2.46 to 4.80) |
| Hospital LOS (day) | 0.72 | (-0.13 to 1.58) |

Notes: ICU use and hospital LOS from a discharged hospital prior to nursing home index admission. All analyses include county and year fixed effects. Standard errors adjusted for heteroscedasticity and clustered at the county level.

ADL = Activities of Daily Living; CFS = Cognitive Function Scale; ICU = Intensive Care Unit; LOS = length of stay.

Appendix Table A2: Changes in the composition of post-acute nursing home patients with ADRD

|  | Estimate (95% CI) | |
| --- | --- | --- |
| Logarithm of the total number of patients | -0.02 | (-0.10 to 0.06) |
| Elixhauser comorbidity index | -0.02 | (-0.17 to 0.14) |

Notes: The total number of patients with ADRD admitted to nursing home directly from hospital discharge. Elixhauser comorbidity index from a discharged hospital prior to nursing home index admission. Aanalyses include county and year fixed effects. Standard errors adjusted for heteroscedasticity and clustered at the county level.

Appendix Table A3: Outcomes of post-acute nursing home care associated with a 10-percentage point increase in Medicare Advantage penetration in a county using varying definitions of ADRD

|  | Main results:  DX and CFS based  Estimate (95% CI) | | DX based only  Estimate (95% CI) | | CFS based only  Estimate (95% CI) | |
| --- | --- | --- | --- | --- | --- | --- |
| Days spent at home | 1.02 | (-0.19 to 2.23) | 0.79 | (-0.29 to 1.86) | 0.62 | (-0.43 to 1.67) |
| Nursing home days | 0.12 | (-1.24 to 1.49) | 0.03 | (-1.31 to 1.37) | 0.97 | (-0.22 to 2.15) |
| Long-stay NH resident (%) | -0.04 | (-0.47 to 0.39) | 0.03 | (-0.39 to 0.46) | 0.23 | (-0.16 to 0.62) |
| Hospital days | -0.05 | (-0.12 to 0.02) | -0.05 | (-0.10 to 0.00) | -0.05 | (-0.11 to 0.02) |
| Hospital readmission (%) | -0.16 | (-0.36 to 0.03) | -0.19 | (-0.36 to -0.02) | -0.24 | (-0.41 to -0.06) |
| Successful discharge to community (%) | 0.73 | (0.33 to 1.12) | 0.56 | (0.23 to 0.90) | 0.62 | (0.28 to 0.96) |
| One-year mortality (%) | -0.16 | (-0.56 to 0.24) | -0.12 | (-0.52 to 0.28) | -0.33 | (-0.69 to 0.04) |
| Admission to NH with star rating ≥ 4 (%) | -0.41 | (-0.96 to 0.14) | -0.17 | (-0.67 to 0.33) | -0.06 | (-0.59 to 0.47) |

Notes: DX based only estimates use only diagnosis information from the MDS to identify patients with ADRD. CFS based only estimates use only cognitive function score information from the MDS to identify patients with ADRD. All estimates are scaled by a 10-percentage point increase in MA penetration in a county. Adjusted for age, race, dual eligibility for Medicare and Medicaid, ADL score, CFS score, Elixhauser comorbidity index, the use of ICU and hospital LOS in a discharged hospital prior to the NH index admission as well as county and year fixed effects. Standard errors are clustered at the county level.
